# Coroners' Fees for Hospital Residents

**Published:** 1909-11-13

**Authors:** T. E. Lister

**Affiliations:** Clayton Hospital, Wakefield


### CORONERS' FEES FOR HOSPITAL RESIDENTS.

DEAR SIR,—I think some useful end might be served by a discussion in your valuable paper of the fact that house surgeons have at present no fees for attending to give evidence at a Coroner's Court. It is high time something was done in this direction. During the last three days I have given evidence at five inquests and done one post-mortem, for which I have not received a shilling.

I am, Sir, yours faithfully,

T. E. LISTER, M.B.

Clayton Hospital, Wakefield.

